# Therapeutic efficacy of liposomal Grb2 antisense oligodeoxynucleotide (L-Grb2) in preclinical models of ovarian and uterine cancer

**DOI:** 10.18632/oncotarget.27667

**Published:** 2020-07-21

**Authors:** Olivia D. Lara, Emine Bayraktar, Paola Amero, Shaolin Ma, Cristina Ivan, Wei Hu, Ying Wang, Lingegowda S. Mangala, Prasanta Dutta, Pratip Bhattacharya, Ana Tari Ashizawa, Gabriel Lopez-Berestein, Cristian Rodriguez-Aguayo, Anil K. Sood

**Affiliations:** ^1^Department of Gynecologic Oncology and Reproductive Medicine, The University of Texas MD Anderson Cancer Center, Houston, TX, USA; ^2^Department of Experimental Therapeutics, The University of Texas MD Anderson Cancer Center, Houston, TX, USA; ^3^Department of Bioinformatics and Computational Biology, The University of Texas MD Anderson Cancer Center, Houston, TX, USA; ^4^Center for RNA Interference and Non-Coding RNA, The University of Texas MD Anderson Cancer Center, Houston, TX, USA; ^5^Department of Cancer Systems Imaging, The University of Texas MD Anderson Cancer Center, Houston, TX, USA; ^6^Bio-Path Holdings, Inc., Bellaire, TX, USA; ^7^Department of Cancer Biology, The University of Texas MD Anderson Cancer Center, Houston, TX, USA

**Keywords:** ovarian cancer, nucleic-acid based therapeutics, therapeutic approaches, uterine cancer

## Abstract

Background: Adaptor proteins such as growth factor receptor-bound protein-2 (Grb2) play important roles in cancer cell signaling. In the present study, we examined the biological effects of liposomal antisense oligodeoxynucleotide that blocks Grb2 expression (L-Grb2) in gynecologic cancer models.

Materials and Methods: Murine orthotopic models of ovarian (OVCAR5 and SKOV3ip1) and uterine (Hec1a) cancer were used to study the biological effects of L-Grb2 on tumor growth. *In vitro* experiments (cell viability assay, Western blot analysis, siRNA transfection, and reverse phase protein array) were carried out to elucidate the mechanisms and potential predictors of tumor response to L-Grb2.

Findings: Treatment with L-Grb2 decreased tumor growth and metastasis in orthotopic models of ovarian cancer (OVCAR5, SKOV3ip1) by reducing angiogenesis and increasing apoptosis at a dose of 15 mg/kg with no effect on mouse body weight. Treatment with L-Grb2 and paclitaxel led to the greatest decrease in tumor weight (mean ± SEM, 0.17 g ± 0.10 g) compared with that in control mice (0.99 g ± 0.35 g). We also observed a reduction in tumor burden after treatment with L-Grb2 and the anti-VEGF antibody B-20 (86% decrease in tumor weight compared with that in controls). Ovarian cancer cells with ErbB2 amplification (OVCAR8 and SKOV3ip1) were the most sensitive to Grb2 downregulation. Reverse phase protein array analysis identified significant dysregulation of metabolites (LDHA, GAPDH, and TCA intermediates) in ovarian cancer cells after Grb2 downregulation.

Interpretation: L-Grb2 has therapeutic efficacy in preclinical models of ovarian and uterine cancer. These findings support further clinical development of L-Grb2.

## INTRODUCTION

Adaptor proteins are essential for signal propagation after receptor tyrosine kinase (RTK) activation [1]. Upon activation, either by ligand binding or protein overexpression, dimerization and stabilization of RTKs occur, resulting in stimulation of tyrosine kinase activation and auto-phosphorylation of tyrosine residues [2, 3]. Phosphotyrosine residues are then used as docking sites for various proteins. Growth factor receptor-bound protein-2 (Grb2) is a 25-kDa adaptor protein that uses its SH2 domain to bind to phosphotyrosine residues in RTKs (EGFR, ErbB2, and VEGF) and its SH3 domains to bind to proline-rich motifs, such as those in the guanine nucleotide exchange factor Son of Sevenless [4–7]. This cascade ultimately leads to activation of RAS/RAF/mitogen-activated protein kinase (MAPK) and phosphatidylinositol 3-kinase (PI3K)/AKT/mTOR signaling pathways critical to tumorigenesis [8, 9]. Because Grb2 is centrally located in signal transduction and is crucially involved in propagation of oncogenic tyrosine kinase signals to downstream mediators, it is an attractive therapeutic target in cancer.

Key adaptor proteins such as Grb2 were previously thought to be undruggable molecular targets. Druggable targets have often been proteins with enzymatically active sites to which small molecules could bind [10]. However, the ability to target previously undruggable targets is evolving. Small-molecule inhibitors rely on intracellular targets or antibodies to inhibit the activity of growth factors, cell surface receptors, and cytokines [11]. The development of nucleic acid interference-based therapeutics has allowed for regulation of gene expression to inhibit elusive targets [12, 13]. Nucleic acid based therapeutics involve a process in which RNA molecules or antisense oligonucleotides (ASOs) inhibit gene expression or translation by neutralizing targeted mRNA molecules [14, 15].

Short interfering RNAs (siRNAs) interact with RNA-induced silencing complexes to block and neutralize targeted mRNAs. After crossing the cell membrane, ASOs target mRNA directly through complementary base pair interactions, in the nucleus or cytosol, thus blocking and neutralizing targeted mRNAs [16, 17]. Both siRNAs and ASOs provide specific, efficient knockdown of gene expression. The antisense approach to gene regulation is a mature method of nucleic acid based therapy with proven efficacy in human trials. Additionally, the ability to modify ASO structure allows for immune system evasion. Due to rapid degradation of RNA in the circulation, delivery of siRNAs and ASOs requires nanoparticle formulations (e. g., liposomes) to carry them to their target tissues [18, 19].

There is a growing need for additional therapeutics in cancer care, specifically in ovarian carcinoma for which the five year survival rate is a dismal 29% for patients presenting with higher stage disease [20]. The high prevalence of molecular alterations in the MAPK and PI3K/AKT/mTOR pathways in ovarian carcinomas suggest that targeting Grb2 could be an important and promising therapeutic opportunity [21–24]. In the present study, we tested the anticancer effects of an ASO that blocks Grb2 protein expression incorporated into a neutral liposome (L-Grb2) in preclinical models of ovarian and uterine carcinoma.

## RESULTS

### Therapeutic efficacy of L-Grb2 in orthotopic models of ovarian cancer

**Figure 1:**
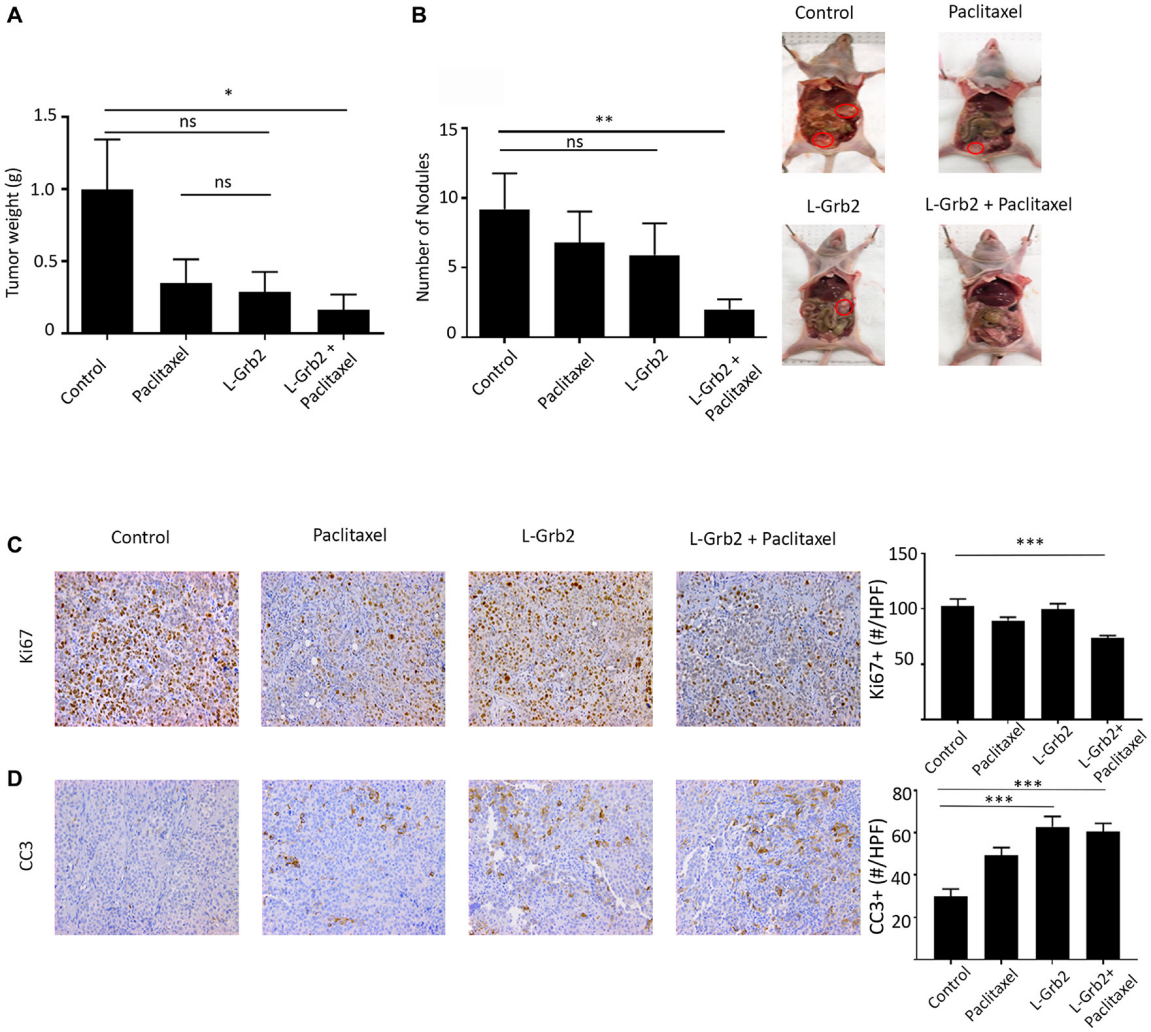
Effects of treatment with L-Grb2 on ovarian tumor growth. (**A**, **B**), Mean tumor weights (A) and numbers of metastatic nodules (B) in mice intraperitoneally inoculated with OVCAR5 cells that received an empty DOPC liposome (control), paclitaxel only (3 mg/kg) weekly, L-Grb2 (15 mg/kg) twice weekly, or a combination of L-Grb2 and paclitaxel beginning 10 days after inoculation (*n* = 9 mice per group). (**C** and **D**), Tumors collected from the mice at the conclusion of *in vivo* therapeutic experiments and tumors were examined using immunohistochemical staining to evaluate the effects of treatment with L-Grb2, paclitaxel, or both in comparison with those of the control treatment on (C) cell proliferation (Ki67 staining) and (D) apoptosis (CC3 staining). Representative images of mice from the four groups taken at 20× magnification are shown at the upper right. The mean numbers of Ki67+ and CC3 + cells per group are shown in the adjoining graphs. Five tumors per group were stained, and five representative images per sample were quantified and used for analysis. Error bars, SEM. All statistical tests were two-sided. Asterisk indicates statistical significance of ^***^ *p* < 0.001, ^**^ *p* < 0.01, ^*^ *p* < 0.05. NS indicates non-significant.

We first performed a L-Grb2 dose-finding experiment using the OVCAR5 ovarian cancer mouse model and measured Grb2 protein expression in harvested tumors at 24 and 72 hours after L-Grb2 administration. Grb2 protein expression was reduced in tumors for up to 72 hours after injection of 15 and 25 mg/kg L-Grb2 (Supplementary Figure 1A). Next, we examined the effects of 15 and 25 mg/kg L-Grb2 on tumor growth *in vivo* using the OVCAR5 model. After intraperitoneal injection of OVCAR5 cells, we gave mice L-Grb2 twice weekly. We observed a reduction in tumor growth at 15 mg/kg, but there was no additive benefit of increasing the L-Grb2 dose. We also saw a reduced number of nodules after treatment with 15 mg/kg L-Grb2. Mouse body weight did not differ markedly between the treatment groups (Supplementary Figure 1B); reduction in tumor weight and nodules was similar between the two treatment groups (Supplementary Figure 1C, 1D). We then moved on to combination therapy with taxane-based therapy since taxanes have combined well with biologically targeted drugs. We first performed a series of experiments to characterize the therapeutic efficacy of L-Grb2 in combination with paclitaxel. In the OVCAR5 model, tumor weight was significantly lower in mice given L-Grb2 and paclitaxel (0.17 g ± 0.10 g, *p* < 0.05) than in control mice (0.99 g ± 0.35 g) (Figure 1A). We also noted a decrease in tumor growth in the mice given L-Grb2 only (0.29 g ± 0.14 g). We observed fewer metastatic nodules in mice given L-Grb2 only or combined with paclitaxel than in control mice given empty DOPC liposome (L-Grb2 only, 5.9 ± 2.9; L-Grb2 and paclitaxel, 2.00 ± 0.72; control, 9.2 ± 2.5, *p* < 0.01) (Figure 1B). We noted no changes in mouse weight and no noticeable changes in mouse mobility during treatment with L-Grb2 (Supplementary Figure 1E).

### Biological effects of L-Grb2 on proliferation and apoptosis

Ovarian tumors harvested from mice were then stained for markers of proliferation (Ki67) and apoptosis (Cleaved-Caspase 3 [CC3]). In the OVCAR5 model, treatment with the combination of L-Grb2 and paclitaxel resulted in the greatest reduction of cellular proliferation as determined via Ki67 staining (mean, 73.50 Ki67+ cells per high-powered field [HPF], *p* < 0.001) when compared to mean number of Ki67+ cells per HPF in control group (102.40) (Figure 1C). In addition, we saw more CC3+ cells in the L-Grb2–alone (mean, 62.82 CC3+ cells per HPF, *p* < 0.001) and combination L-Grb2 and paclitaxel (mean, 60.55 CC3+ cells per HPF, *p* < 0.001) groups than in the control (mean, 29.95 CC3+ cells per HPF) and paclitaxel-alone (mean, 49.30 CC3+ cells per HPF) groups (Figure 1D). The reduction in number of proliferative cells and increase in apoptotic cells was significant in the combination group compared to control groups.

### Effect of Grb2 downregulation in ovarian cancer cells *in vitro*

**Figure 2:**
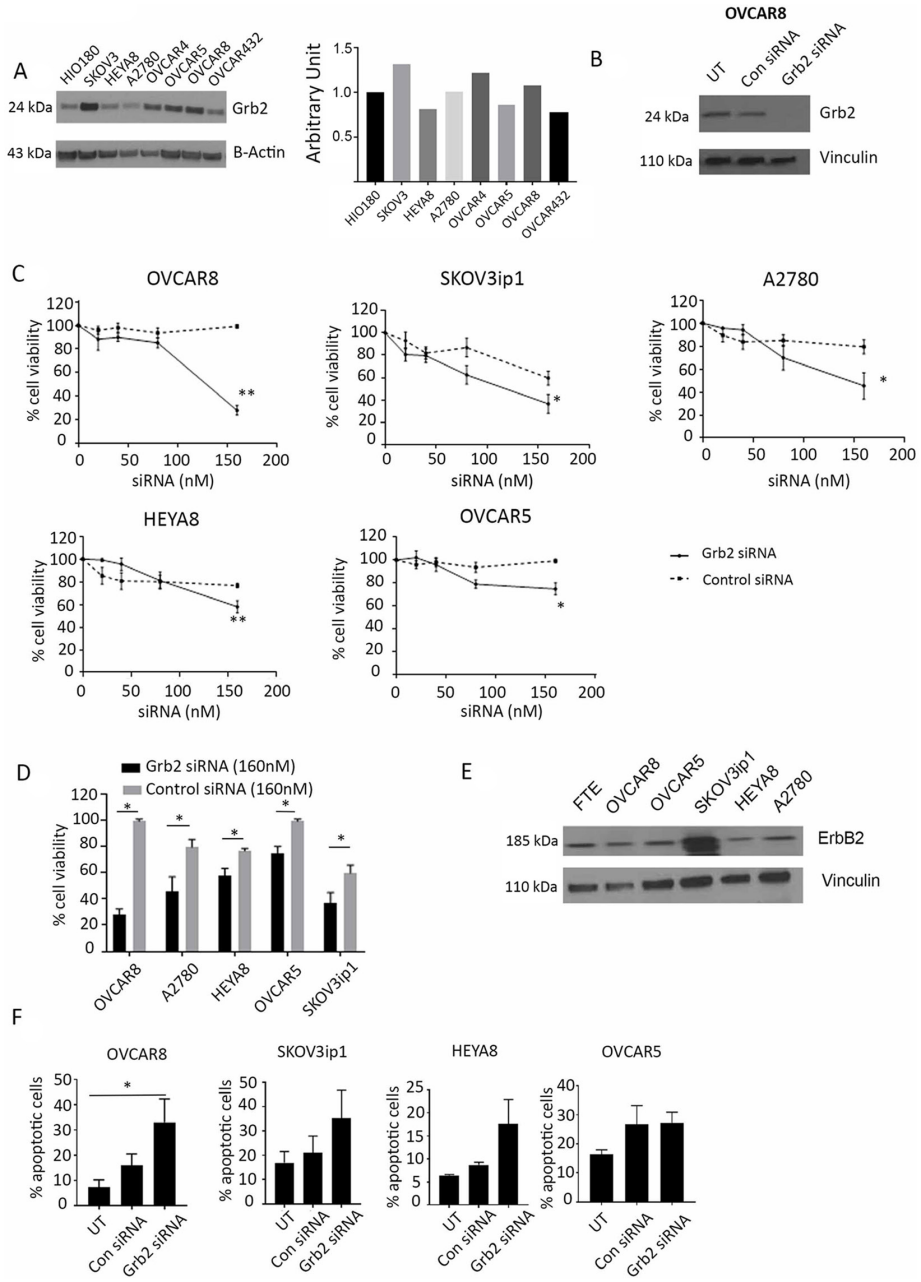
Effect of Grb2 downregulation on ovarian cancer cell lines. (**A**) Western blot analysis of Grb2 expression in a panel of ovarian cancer cell lines compared with that in the non-transformed epithelial ovarian cell line HIO180. The adjoining graph shows their expression compared with that in HIO180. (**B**) Western blot analysis of Grb2 expression in OVCAR8 cells 72 hours after siGrb2 (Grb2 siRNA) cells compared with that in untreated (UT) and siControl (Con siRNA) cells. (**C**) OVCAR8, SKOV3ip1, A2780, HEYA8, and OVCAR5 cell lines were transfected with siGrb2 or siControl at increasing concentrations. An alamarBlue assay of the cells was performed 72 hours after transfection to determine their percent viability, which is shown in the graphs. The data represent averages of triplicate measurements. (**D**) The percent viability of the five cell lines shown in panel C, 72 hours after transfection of siGrb2 and siControl at 160 nM. (**E**) Western blot analysis of erbB2 expression in a panel of ovarian cancer cell lines compared to fallopian tubal epithelium (FTE). (**F**) Results of an annexin V assay performed to determine the number of apoptotic untreated (UT), siControl-transfected (100 nM), and siGrb2-transfected (100 nM) ovarian cancer cells. The assay was performed 72 hours after transfection. Error bars, SEM. All statistical tests were two-sided. Asterisk indicates statistical significance of ^**^ *p* < 0.01, ^*^ *p* < 0.05.

We measured the baseline expression of Grb2 in a panel of seven ovarian cancer cell lines and compared it with that in the non-transformed ovarian cell line HIO180 (Figure 2A). We then transfected the cells with 100 nM of siControl or siGrb2 to downregulate Grb2 protein expression (Figure 2B). After observing decreased protein expression of Grb2 in all cell lines, we assessed the effect of Grb2 downregulation on three cell lines with high Grb2 protein expression (OVCAR8, OVCAR5, and SKOV3ip1) and two with low Grb2 protein expression (HEYA8 and A2780) using a cell viability assay. OVCAR8 and SKOV3ip1 cells were the most sensitive to Grb2 downregulation (Figure 2C and 2D). We characterized the cell lines according to their mutation status and found that cell lines with erbB2 mutation or amplification were the most sensitive to Grb2 downregulation (Table 1). Increased erbB2 protein expression was confirmed on western blot analysis (Figure 2E).

**Table 1:** Mutation status of the ovarian cancer cell lines used in the study [57, 58]

Cell line	Gene				
	ErbB2	PIK3CA	PTEN	KRAS	BRAF
OVCAR8	Mut	WT	WT	Mut	WT
A2780	WT	Mut p. E365K	Mut p. KGR128del	WT	Mut
OVCAR5	WT	WT	WT	Mut pG12V	WT
HeyA8	WT	WT	WT	Mut	Mut
SKOV3ip1	Amp	Mut p. H1047R	WT	WT	WT

Abbreviations: Amp, amplification; Mut, mutant; WT, wild-type.

Because of the inhibition of ovarian tumor growth and increased apoptosis in *in vivo* tumor specimens, we next examined the *in vitro* effects of Grb2 downregulation by siRNA on the ovarian cancer cell lines described above. An annexin V assay demonstrated increased apoptosis after Grb2 downregulation in all cell lines, with the greatest effects seen in the OVCAR8 cells (siGrb2, 33.0% apoptotic; untreated, 7.3% apoptotic, *p* < 0.05) and SKOV3ip1 cells (siGrb2, 35.16% apoptotic; untreated, 16.98% apoptotic) (Figure 2F). Next, we examined the effect of Grb2 downregulation on ovarian cancer cell proliferation. We observed no effect on the number of proliferative OVCAR8 cells at 72 hours (siControl, 34.95 ± 0.94; siGrb2, 37.94 ± 1.54) (Supplementary Figure 2A). We also observed no change in the number of colonies formed in OVCAR5 cells in a colony formation assay (siControl, 1014 ± 121; siGrb2, 848 ± 53). However, the quantified colonies were smaller in the siGrb2-exposed group than in the controls (mean ± SEM colony area: siGrb2, 1.290 ± 0.175 mm^2^; siControl, 2.45 ± 0.50 mm^2^) (Supplementary Figure 2B). These results corroborated our *in vivo* findings that Grb2 downregulation due to treatment with L-Grb2 leads to an increase in the number of apoptotic cells. We found no effects on cell cycle progression after Grb2 downregulation (Supplementary Figure 3A). Finally we performed invasion assay at varying time points. At 72 hours after transfection, we found cells treated with siGrb2 were largely apoptotic. At 48 hours, cells treated with siGrb2 were found to have no effect on invasive potential (Supplementary Figure 3B).

### Pathway analysis after Grb2 downregulation on ovarian cancer cells

**Figure 3:**
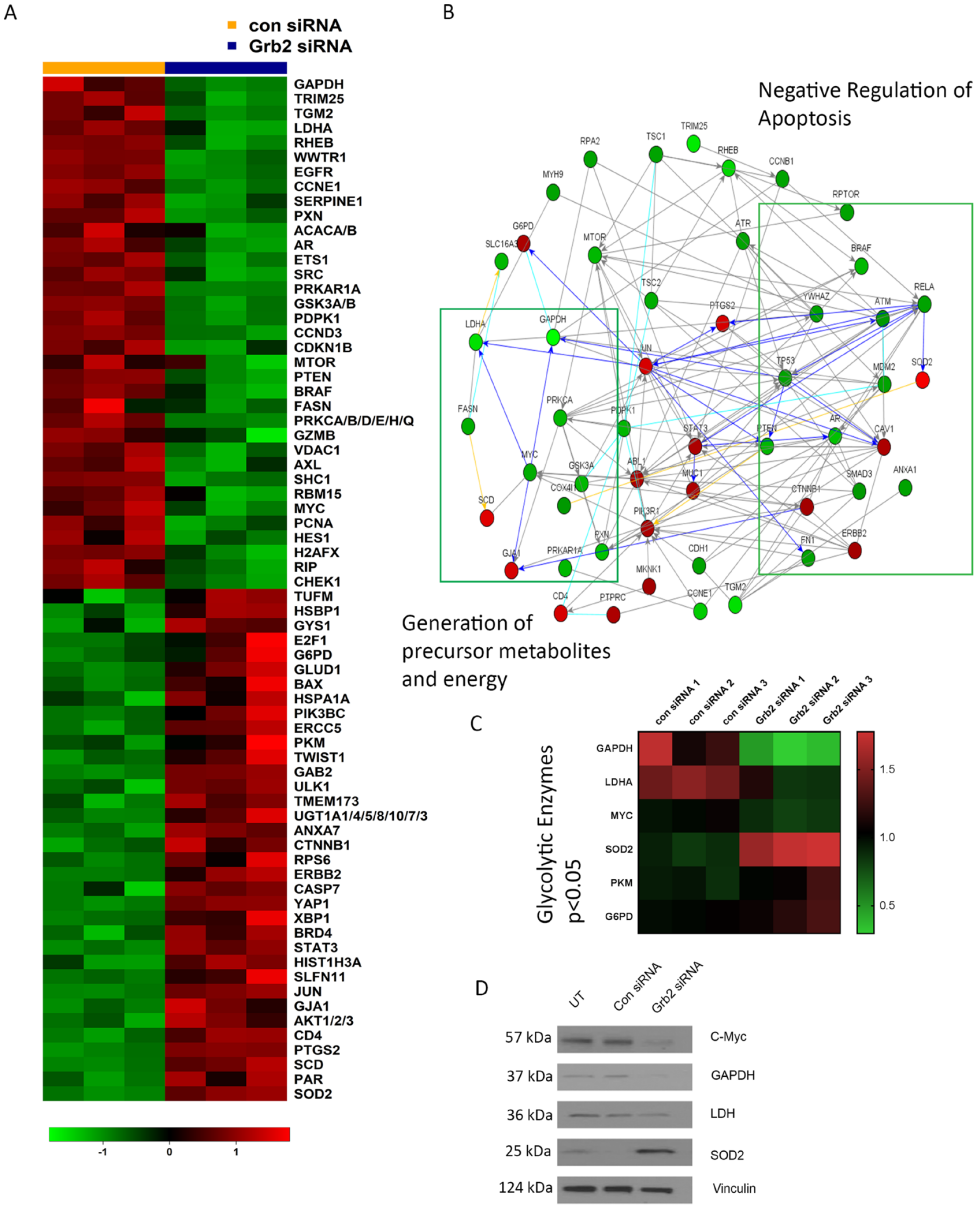
Differential expression of proteins OVCAR8 ovarian cancer cells after Grb2 downregulation as detected by RPPA. (**A**) Heat map of proteins whose expression differed significantly between siGrb2- and siControl-transfected groups (*p* < 0.05). (**B**) Networks generated after Grb2 downregulation using NetWalker software. Fold changes in protein expression were calculated on the basis of NormLog2 expression differences between the siControl- and siGrb2-transfected cells. (**C**) Heat map of glycolytic enzymes whose expression differed between siGrb2- and siControl-transfected groups (*p* < 0.05). (**D**) Western blot analysis of glycolytic enzymes after siGrb2 transfection.

**Figure 4:**
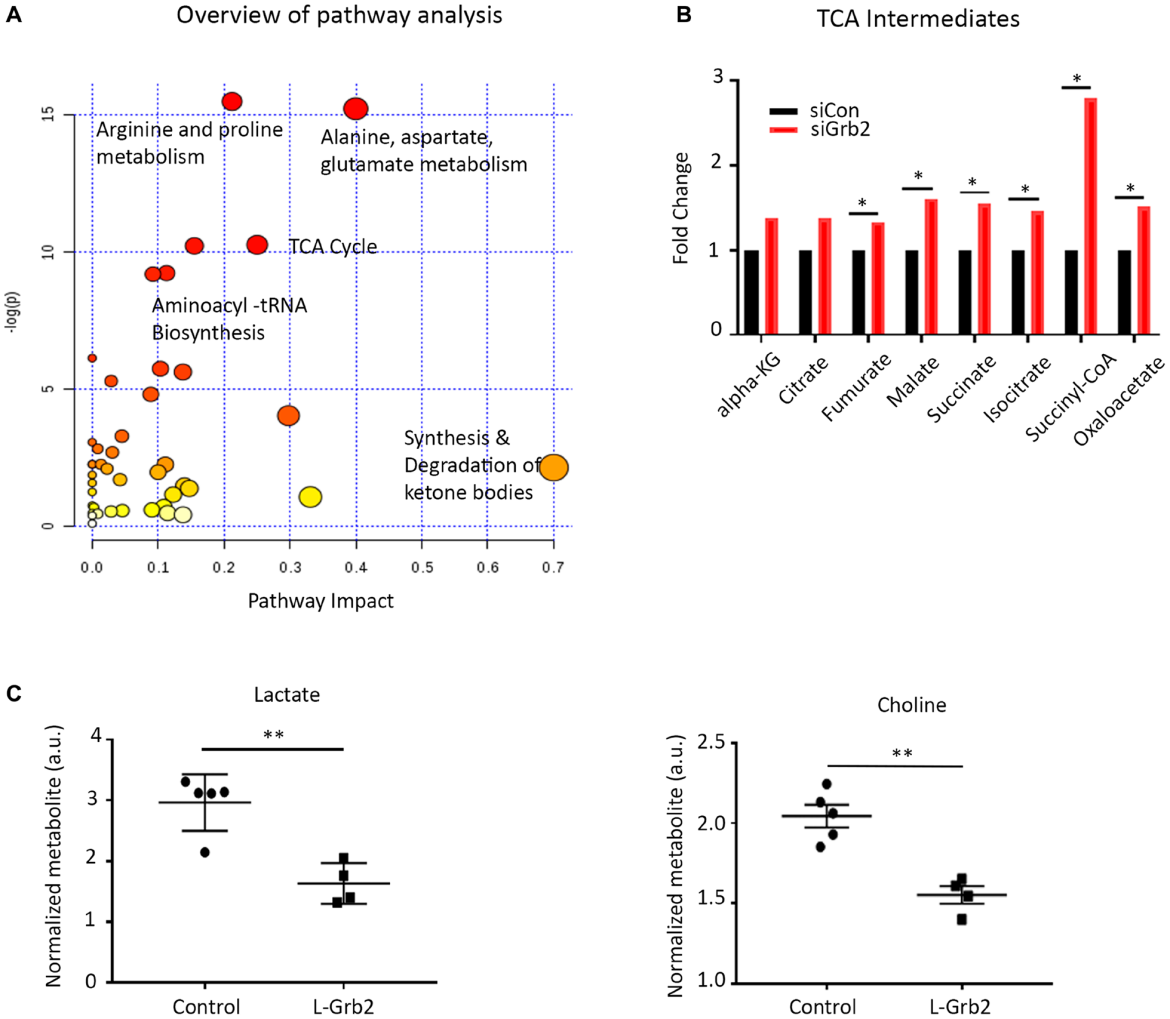
Effect of Grb2 downregulation on metabolite levels in ovarian cancer cells. (**A**) Metabolites analyzed using the pathway analysis module of MetaboAnalyst. TCA, tricarboxylic acid; tRNA, transfer RNA. (**B**) TCA metabolite levels in OVCAR8 ovarian cancer cells after siGrb2 and siControl transfection. (**C**) Effect of Grb2 downregulation on lactate and choline levels in ovarian tumors. Mass spectroscopy was used to quantify metabolite levels in OVCAR5 tumors collected at the conclusion of an *in vivo* therapeutic experiment from control mice and mice given L-Grb2 based monotherapy (*n* = 5). Error bars, SEM. All statistical tests were two-sided. Asterisk indicates statistical significance of ^**^ *p* < 0.01, ^*^ *p* < 0.05.

Next, to understand the broader downstream effects of Grb2 inhibition on ovarian cancer cells, we conducted a RPPA analysis with OVCAR8 cells (Figure 3A). To identify pathways in these cells affected by Grb2 downregulation after transfection with siGrb2, we used the NetWalker software program (Figure 3B). Networks significantly affected by Grb2 downregulation included *Generation of precursor metabolites and energy* (downregulated) and *Negative regulation of apopt*osis (downregulated) (Supplementary Table 1). Additionally, there was a downregulation of insulin receptor signaling (Supplementary Table 1) and glycolytic metabolites (Figure 3C). We confirmed the protein expression of glycolytic enzymes and marker of mitochondrial stress, superoxide dismutase 2 (SOD2), by western blot (Figure 3D). It is well documented that rapidly proliferating tumor cells rely on aerobic glycolysis in a phenomenon referred to as the Warburg effect [25–27]. Deregulated c-MYC, HIF1α and growth signaling lead to induction of glycolytic enzymes and inhibition of pyruvate oxidation in mitochondria [28–30]. Disrupting the Warburg effect to shunt cancer metabolism to oxidative phosphorylation, subsequently increases oxidative stress and triggers apoptosis [31–33]. Therefore, based on our RPPA and pathway analysis, we hypothesized that Grb2 downregulation was leading to a disruption in the Warburg effect, shunting metabolism to the tricarboxylic acid (TCA) cycle. To confirm this, we performed metabolomics analysis of OVCAR8 cells with Grb2 downregulation. Specifically, we compared the metabolite levels in OVCAR8 cells transfected with siControl to those cells transfected with siGrb2. We analyzed a total of 295 metabolites and found that 61 of them were significantly dysregulated after Grb2 downregulation (Supplementary Figure 4A). We then performed metabolite set enrichment analysis of the relative concentrations of metabolites with significant differences between OVCAR8 cells with Grb2 downregulation and control cells to identify biological patterns (Supplementary Figure 4B). Additionally, we used the metabolomic pathway analysis module of the MetaboAnalyst software program to identify the pathways most affected by Grb2 downregulation. Dysregulated metabolites lead to enrichment of amino acid metabolism and the TCA cycle (Figure 4A). Because pathway analysis could only provide associations between metabolites and pathway regulation, we went back to our original data to quantify metabolites specific to the TCA cycle. We found an increase in TCA intermediates, fumurate, malate, succinate, isocitrate, succinyl-coA and oxaloacetate in cells transfected with siGrb2 (*p* values < 0.05 when compared to siControl) (Figure 4B). Finally, we corroborated these results with our harvested *in vivo* tumors through NMR spectroscopy. After tumors were analyzed, metabolite levels between L-Grb2 and empty DOPC treated tumors were compared. We found substantially lower lactate and choline levels in the L-Grb2 monotherapy group of tumors than in the control group (Figure 4C, Supplementary Figure 5). Based on these findings we concluded that Grb2 downregulation leads to a disruption of the Warburg effect through a decrease in lactate dehydrogenase A (LDHA). This subsequently increases oxidative phosphorylation, and mitochondrial stress as seen through an increase in superoxide dismutase 2 (SOD2).

### Antiangiogenic effects of L-Grb2 and B-20 in ovarian tumors

The vascular endothelial growth factor (VEGF) signaling pathway plays a pivotal role in angiogenesis, thus several strategies have been designed to target VEGF signal transduction [34, 35]. The effects of VEGF are mediated by two receptor tyrosine kinases, VEGFR-1 and VEGFR-2, which require Grb2 for signaling [36]. To investigate the relationship between Grb2 and angiogenesis through VEGF signaling, we examined TCGA data. We found Grb2 expression correlates strongly with VEGFR-1 (R = 0.61, *p* < 0.001), VEGFR-2 (R = 0.62, *p* < 0.001), expression and pro-angiogenic genes VE-Cadherin (R = 0.43, *p* < 0.001), and PECAM1 (R = 0.75, *p* < 0.001), (Supplementary Figure 6A). Given these findings, we hypothesized that Grb2 downregulation may lead to a decrease in angiogenesis and thus work well with anti-angiogenic therapy. To test this, we performed a cell viability assay and found a decrease in the number of viable RF-24 endothelial cells after Grb2 downregulation (*p* < 0.05) (Supplementary Figure 6B). We then tested the effect of Grb2 downregulation on RF-24 cells *in vitro* using an endothelial cell tube formation assay (Supplementary Figure 6C). We observed a decrease in endothelial cell viability with Grb2 downregulation and a corresponding decrease in the number of tubes formed by RF-24 cells (mean [± SEM], 19 ± 1 tubes for siGrb2-transfected cells and 76 ± 6 tubes for siControl-transfected cells, *p* < 0.05).

**Figure 5:**
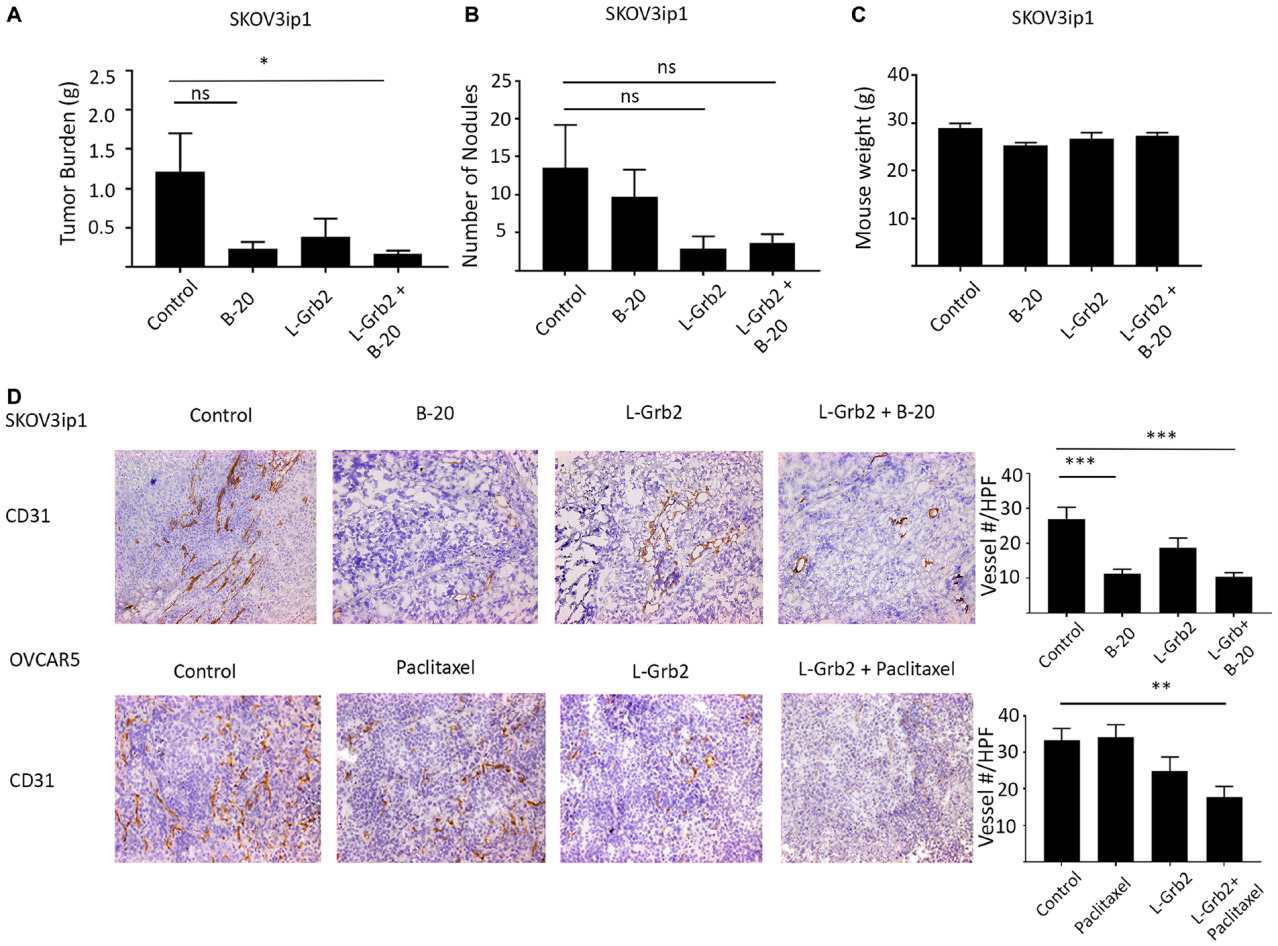
*In vivo* effects of treatment with L-Grb2 in combination with anti-angiogenic therapy in an ovarian tumor model. (**A**–**C**) Mean tumor weights in (A), numbers of metastatic nodules in (B), and body weights of (C) mice intraperitoneally inoculated with SKOV3ip1 cells that received control treatment, B-20 only (6.25 mg/kg) twice weekly, L-Grb2 only (15 mg/kg) twice weekly, or a combination of B-20 and L-Grb2 beginning 10 days after inoculation (*n* = 9 mice per group). (**D**) Tumors collected from the SKOV3ip1 and OVCAR5 models at the conclusion of *in vivo* therapeutic experiments were subjected to immunohistochemical staining for CD31 to evaluate the effects on tumor vessel number of treatment with L-Grb2, B-20, or both compared with the control treatment. Representative staining images taken at 20× magnification are shown. The mean CD31+ cell numbers per group are shown in the adjoining graphs. Five tumors per group were stained, and five representative images per sample were quantified for analysis. Error bars, SEM. All statistical tests were two-sided. Asterisk indicates statistical significance of ^***^ *p* < 0.001, ^**^ *p* < 0.01, ^*^ *p* < 0.05. NS indicates non-significant.

Next, we evaluated the effects of L-Grb2 on angiogenesis *in vivo*. First, we investigated the effects of treatment with L-Grb2 in combination with B-20 (an anti-VEGF antibody) in SKOV3ip1 cells. We also observed substantial reductions in tumor weight in all groups of mice, with an 86% decrease in tumor weight in mice given the combination of L-Grb2 and B-20 (mean ± SEM, 0.16 g ± 0.05 g versus 1.21 g ± 0.49 g in the control group, *p* < 0.05). The number of tumor nodules was decreased in all groups. There were no significant differences in mouse weights in the treatment groups (Figure 5A–5C). We then stained tumors harvested from the study mice for CD31. We found decreased number of vessels in all treatment groups, as the mean (± SEM) vessel numbers were 26.97 ± 3.37 in the control group, 11.28 ± 1.29 in the B-20–only group, 18.67 ± 2.84 in the L-Grb2–only group, and 10.38 ± 1.19 in the combination L-Grb2 and B-20 group (*p* < 0.001 compared to control) (Figure 5D). We also stained OVCAR5 tumor sections for CD31 and found a significantly lower mean (± SEM) number of vessels in mice given the combination of L-Grb2 and paclitaxel (17.78 ± 3.46, *p* < 0.01 compared to control) than in the L-Grb2–only (24.88 ± 3.88), control (33.33 ± 3.25), and paclitaxel-only (34.11 ± 4.75) groups (Figure 5D).

### Effect of Grb2 downregulation in uterine cancer cells *in vitro* and in orthotopic models of uterine cancer

Next we sought to determine the role of Grb2 in uterine cancer models. Uterine carcinoma is the most common gynecologic malignancy in the United States, for which the number of available therapies is limited [37]. Molecular characterization of endometrial tumors has demonstrated that PIK3CA mutations, PTEN loss, and PI3K and KRAS activation are key events in carcinogenesis [38, 39]. In addition to these critical mutations, ErbB2 is amplified in 17–33% of carcinosarcomas, and uterine serous carcinomas [40, 41]. Given our findings in ErbB2 mutated or amplified ovarian cancer cell lines, we hypothesized that Grb2 was a critical mediator of oncogenic signaling in uterine carcinomas. Baseline expression of Grb2 protein in a panel of uterine cancer cell lines (MFE 319, Ishikawa, Hec1a and KLE) is reported in Supplementary Figure 7A. We focused on uterine cell line Hec1a known to have erbB2 amplification. Cell viability assay on Hec1a verified sensitivity to Grb2 downregulation compared to control siRNA (Supplementary Figure 7B). RPPA analysis of Hec1a cells transfected with siControl or siGrb2 (Supplementary Figure 7C) revealed that networks significantly downregulated after siGrb2 transfection include AMPK signaling, PI3K/AKT signaling and insulin receptor signaling (Supplementary Figure 7D). Next, we evaluated the effects of L-Grb2 as monotherapy and in combination with paclitaxel and bevacizumab in the Hec1a model. Ten days following inoculation of Hec1a cells into the right uterine horn, treatment with L-Grb2, paclitaxel and bevacizumab was initiated. Tumor growth was significantly decreased in all groups compared to control (mean ± SEM; control: 1.67 g ± 0.30 g; paclitaxel and bevacizumab only: 0.74 ± 0.30 g; L-Grb2 only: 1.18 ± 0.37 g, and paclitaxel, bevacizumab and L-Grb2 group: 0.38g ± 0.25 g) (Supplementary Figure 8A). The most significant reduction in tumor was seen in the triple combination group, with a 77% decrease in tumor burden compared to control (*p* < 0.0001). There was a significant reduction in number of tumor nodules in the triple combination group compared to control (mean ± SEM; control: 13.3 ± 4.3 nodules; triple combination group 3.6 ± 4.3 nodules, *p* < 0.05) (Supplementary Figure 8B). There was no effect on mouse weight in any treatment group (Supplementary Figure 8C).

## DISCUSSION

The key findings of our study include the therapeutic efficacy of L-Grb2 in preclinical ovarian and uterine cancer models due to increased cancer cell apoptosis and reduced tumor angiogenesis. Also, we observed additive antitumor effects of L-Grb2 when given with paclitaxel in ovarian tumor models. Additionally, L-Grb2 potentiated the effects of the anti-angiogenic therapeutic B-20 in the models. Finally, we found ovarian cancer cell lines with ErbB2 mutations or overexpression to be particularly sensitive to Grb2 downregulation. This correlated with a decrease in metabolite levels related to glycolysis (LDHA, GAPDH) in cell lines after Grb2 downregulation or L-Grb2–based treatment.

Downregulating Grb2 protein expression via treatment with L-Grb2 is a promising molecular therapy for ovarian and uterine cancer using a target previously thought to be undruggable. Given the heterogeneity and large number of molecular alterations in ovarian tumors, identifying targets that are of therapeutic benefit is challenging. However, the use of molecular pathways to develop small-molecule inhibitors and individualize treatment strategies remains a promising avenue to improve ovarian cancer patient survival. Grb2 plays a central role in RTK signaling, particularly EGFR and ErbB2 signaling. About 11% of ovarian tumors have ErbB2 amplification, whereas EGFR overexpression is found in up to 28% and amplified in up to 20% of ovarian cancers [42–44]. More importantly, overexpression of EGFR and ErbB2 has been associated with poor survival in gynecological cancer patients [45]. This is likely due to their association with the RAS/RAF/MAPK and PI3K/PTEN/AKT/mTOR pathways, which are activated in 70% and 50% of ovarian cancer cases, respectively [42]. The high prevalence of these molecular alterations in ovarian cancer patients represents an important therapeutic opportunity using L-Grb2.

Given the high rate of recurrence of and poor prognosis for ovarian cancer, therapies that may prolong survival are needed. Targeted and biological therapies for ovarian cancer currently in development or in use include anti-angiogenic drugs, PARP inhibitors, immunotherapy, and small-molecule inhibitors of signaling pathways [46, 47]. Whereas only bevacizumab and PARP inhibitors are approved by the U. S. Food and Drug Administration as targeted therapy for ovarian cancer, the number of small-molecule inhibitors in clinical development is increasing [48]. MAPK and PI3K/AKT/mTOR are important cellular signaling pathways involved in proliferation, tumorigenesis, cell survival, angiogenesis, and protein synthesis [49]. Small-molecule inhibitors, including temsirolimus (a TOR complex 1 inhibitor), pictilisib (a PI3K inhibitor), and selumetinib (a MAPK kinase inhibitor), have had varying levels of efficacy in ovarian cancer patients [21, 50–52]. The MAPK and PI3K/AKT/mTOR pathways converge at several points; therefore, dual or upstream blockade of them may have synergistic effects and overcome tumor resistance to current small molecule inhibitors in development.

Researchers recently assessed the clinical activity of L-Grb2–based therapeutic BP1001 (Bio-Path Holdings, Inc.) in patients with refractory or relapsed acute myeloid leukemia in a phase 1 trial. They found that targeting Grb2 is particularly promising in treatment of leukemia given the large number of activating mutations of tyrosine kinases in leukemia cells. BP1001 was well tolerated and had anti-leukemic activity when delivered as monotherapy and in combination with cytarabine. The investigators did not identify a maximum tolerated dose of BP1001, and the most common grade 3-4 adverse events were cardiopulmonary disorders (25 [64%] of 39 patients) and fever and infections (17 [44%]) [53]. Enrollment in a phase 2 study of BP1001 of patients with previously untreated acute myeloid leukemia is underway.

Herein, we report that L-Grb2 has promising antitumor activity in preclinical models of ovarian and uterine carcinoma. Whereas the evidence of L-Grb2’s activity against hematological malignancies is promising, whether it is active in clinical trials against solid tumors has yet to be tested. Therapies targeting the ErbB2 receptor have had limited success in ovarian cancer, but L-Grb2 may be a better target given its status as an important converging point for cancer cell signaling pathways.

## MATERIALS AND METHODS

### Cell line maintenance and siRNA transfection

The ovarian cancer cell lines OVCAR8, HeyA8, A2780ip1, and SKOV3ip1 were maintained in RPMI 1640 medium supplemented with 10% FBS and 0.1% gentamicin sulfate (Gemini Bioproducts). OVCAR5 ovarian cancer cells and KLE uterine cancer cells were maintained in Dulbecco’s modified Eagle’s medium with 10% FBS and 0.1% gentamicin sulfate. RF-24 endothelial cells were maintained in Dulbecco’s modified Eagle’s medium (MEM) supplemented with pyruvate, amino acids, and penicillin/streptomycin. Uterine cancer cell lines MFE319 and Ishikawa were maintained in MEM supplemented with 10% FBS and 0.1% gentamicin sulfate. Hec1a uterine cancer cells were maintained in McCoy’s 5A supplemented with 10% FBS and 0.1% gentamicin sulfate. All of these cells were cultured at 37°C using a 5% CO_2_ incubator. Cell line authentication was performed by the Characterized Cell Line Core at The University of Texas MD Anderson Cancer Center. Mycoplasma testing of the cells was performed using an ATCC Universal Mycoplasma Detection Kit. All *in vitro* experiments were conducted with 80% confluent cultures and fewer than 20 passages. Ovarian cancer cells were transfected with Grb2 siRNA or control siRNA. All siRNA sequences were purchased from Sigma-Aldrich (SASI_Hs01_00129586). Cells were seeded in six-well plates at a density that yielded 50–60% confluence after 24 hours of plating (100,000 to 150,000 cells/well). The next day, 1.3 μL (100 nM) of siGrb2 sequences were mixed at a 1:3 ratio with Lipofectamine 2000 (lot #1774775; Invitrogen) prepared in serum-free medium for 20 minutes. The transfection complex was added to cells with serum-free medium. Cells were incubated with the siRNA/Lipofectamine 2000 complex for 4 hours in a 37°C, 5% CO_2_ incubator and refreshed with complete medium after 4 hours. Cells were then harvested for Western blot analysis to verify Grb2 knockdown. For transfection in 96-well plates, cells were plated at a density of 5,000–7,000 cells per well in technical replicates in six wells per siRNA sequence. The next day, cells were transfected with 0.21 μL of siRNA in serum-free media and incubated for 4 hours in a tissue culture incubator as described above. Cells were then re-fed with complete media and subjected to alamarBlue viability assays.

### Cell viability assays

Cell viability assays were performed by testing cells’ ability to reduce alamarBlue (Bio-Rad). Ovarian cancer cell lines were seeded in a 96-well plate and transfected the next day with increasing concentrations of siControl or siGrb2. After 72 hours, cells were incubated with 10% alamarBlue for 4 hours at 37°C. The absorbance at 540 nm was then recorded.

### Immunoblotting

After siRNA transfection, cells were harvested and lysed with RIPA buffer (1% Triton X-100, 25 mM Tris, 150 mM NaCl, 0.1% SDS, 0.5% sodium deoxycholate) supplemented with fresh protease and phosphatase inhibitors (TC260670 and TJ272575; Thermo Fisher Scientific). Protein quantification was performed using a BCA Protein Assay Kit (#23235; Thermo Fisher Scientific) following the manufacturer’s protocol. Thirty micrograms of cell lysates was loaded onto SDS-PAGE gels. After separation, proteins were transferred to nitrocellulose membranes and blocked with 5% nonfat dry milk (#AB10109-0100; AmericanBio) in TBS-T (0.1% Tween-20) for 1 hour at room temperature. After blocking, indicated antibodies diluted in 5% milk in TBS-T were placed on membrane overnight at 4°C. The next day, the membranes were washed three times with TBS-T for 10 minutes with light agitation. Afterward, a species-specific secondary antibody was placed on membrane for 2 hours at room temperature. The membranes were then washed three times in TBS-T and finally developed using Western Lightning Plus-ECL (#NEL105001EA; PerkinElmer) on X-ray film (#F-BX57; Phoenix Research Products). For re-probing of Western blots, membranes were stripped using Restore PLUS Western Blot Stripping Buffer (#46430; Thermo Fisher Scientific), re-blocked with 5% milk in TBS-T, and incubated with a primary antibody. The antibody dilutions were as follows: anti-Grb2, 1:1000 (#3972; Cell Signaling Technology); anti-ErbB2, 1:100 (2242S; Cell Signaling Technology), anti-vinculin, 1:2000 (V9131, lot #118M4777V; Sigma), anti-β-actin, 1:2000 (127M4866V; Sigma), anti-GAPDH, 1:1000 (5174; Cell Signaling Technology), anti-LDHA, 1:1000 (3582; Cell Signaling Technology), anti-c-MYC, 1:1000 (5605; Cell Signaling Technology) and anti-SOD2 (13141; Cell Signaling Technology).

### Murine orthotopic models of ovarian and uterine carcinoma

All mice used in the study were 8-12 weeks old at the beginning of the experiments. For all animal experiments, cells were harvested using trypsin-EDTA, neutralized with FBS-containing media, and re-suspended in Hank’s balanced salt solution (Gibco) before injection into mice. To generate ovarian carcinoma models, OVCAR5 cells (1 × 10^6^ in 200 μL of Hank’s balanced salt solution) and SKOV3ip1 cells (1 × 10^6^ in 200 μL of Hank’s balanced salt solution) were injected into mice intraperitoneally. To generate the uterine carcinoma model, Hec1a cells (4 × 10^6^ in 25 μL of Hank’s balanced salt solution) were injected into the right uterine horn of mice. Mice were given paclitaxel (35 μg per mouse) once weekly or B-20-4.1.1 (VEGF: 5563, Lot #71943-30, Genentech) (6.25 mg/kg) or bevacizumab (NDC 50242-061-01, Genentech, Inc.) (5 mg/kg) twice weekly via intraperitoneal injection. Furthermore, empty DOPC liposomes or L-Grb2 (Lot # BP1001-002; Bio-Path Holdings, Inc.) were injected intravenously via the tail vein at a dose of 15 mg/kg twice weekly. Once mice in any group became moribund, all mice were sacrificed. Tumors were harvested from the mice and weighed, and the numbers of nodules and tumor weights were recorded. Tumor tissue was preserved and fixed in formalin for paraffin embedding, frozen in optimal cutting temperature medium to prepare frozen slides, or snap-frozen for lysate preparation.

### Immunohistochemistry

Harvested tumor samples were embedded in paraffin blocks and sectioned by the MD Anderson Research Histology Core Laboratory. Paraffin-embedded tissue samples were used to stain for Ki67 (RB-9043-P1; Thermo Fisher Scientific) and cleaved caspase-3 ([CC3]; 9661; Cell Signaling Technology). Briefly, sections of the samples were deparaffinized sequentially in xylene and decreasing concentrations of ethanol prior to rehydration and transfer to PBS. For CC3 antigen retrieval slides were placed in a vegetable steamer (Hamilton Beach) in sodium citrate (pH 6) buffer for 25 minutes. Antigen retrieval for staining for Ki67 was performed in Diva Decloaker solution (#DV2004MX; Biocare Medical). Endogenous tissue peroxidase activity was quenched with 3% hydrogen peroxide in 100% methanol for 12 minutes. Slides were then washed and blocked with 5% goat serum in PBS for 1 hour at room temperature. A primary antibody was then diluted in 5% goat serum in PBS overnight at 4°C in a humidified chamber. Ki67 was diluted at 1:200, whereas CC3 was diluted at 1:100. Slides were then washed three times with PBS. CC3 stained slides were incubated with a biotinylated anti-rabbit antibody (#GR602H; Biocare Medical) for 20 minutes at room temperature. Next, slides were washed three more times with PBS and incubated for 20 minutes with a streptavidin-horseradish peroxidase label (#HP604H; Biocare Medicare). For Ki67 staining, slides were incubated with a secondary anti-rabbit antibody conjugated to horseradish peroxidase (#111-036-047; Jackson ImmunoResearch) diluted in 5% goat serum in PBS at a 1:500 dilution for 1 hour at room temperature. CD31 staining of frozen sections was also performed. Sections were fixed in cold acetone for 15 minutes, washed with PBS, blocked with 5% goat serum in PBS, and incubated with a rat monoclonal anti-mouse CD31 antibody (1:200, 553370; Pharmingen) overnight at 4°C. The next day, slides were washed with PBS, and an appropriate horseradish peroxidase-conjugated secondary antibody was placed on them for 1 hour at room temperature. After secondary antibody incubation, slides were again washed with PBS, briefly washed with PBS containing Brij 35 (#858366; Sigma-Aldrich), and placed in 3,3′-diaminobenzidine (#750118; Thermo Fisher Scientific). Upon color change, slides were rinsed in Milli-Q water and counterstained with hematoxylin (#GHS316; Sigma-Aldrich) for 13 seconds, rinsed in water again, and left to dry. Slides were then mounted with coverslips using Permount medium (#SP15-100; Thermo Fisher Scientific). Slides were imaged using a Leica DM4000 B LED microscope. For quantification of tumor specimens, five random high-power field (HPF) photographs of each slide were taken, and stained cells were counted manually.

### EdU incorporation assay, annexin V staining, and cell-cycle assay

Ovarian cancer cells were plated in technical duplicates per experiment in six-well plates at a density of 50,000–100,000 cells per well. The next day, cells were transfected with siRNA as described above. SiGrb2 and siControl cells were harvested 72 hours after transfection. Harvested cells were then pulsed with EdU for 2 hours and processed using a Click-iT Plus EdU Alexa Fluor 488 Flow Cytometry Assay Kit (#C10632; Thermo Fisher Scientific) following the manufacturer’s protocol. For annexin V staining of ovarian cancer cells after transfection a BD Biosciences FITC Annexin V Apoptosis Detection Kit I (#556547) was used according to the manufacturer’s protocol. After annexin V analysis, cells were stained with 4′,6-diamidino-2-phenylindol) for use in cell-cycle analysis. For flow cytometry analysis and data collection, a Beckman Coulter Gallios Flow Cytometer was used.

### Colony formation assay

Ovarian cancer cells were plated in technical duplicates per experiment in six-well plates at a density of 1000 cells per well. Twenty-four hours after seeding, cells were transfected with either siControl or siGrb2 using methods described previously. Cells were left to grow in a tissue culture incubator for 7–10 days. Afterward, the cells were washed two times with ice-cold PBS and fixed with ice-cold 100% methanol for 10 minutes. After 10 minutes, the methanol was discarded, and the cells were stained with a crystal violet solution (0.5% crystal violet with 20% methanol in Milli-Q water; Sigma-Aldrich) for 10 minutes at room temperature. Crystal violet was then removed, and the cells were washed with deionized Milli-Q water three times and left to dry at room temperature.

### Invasion assay

Invasion assays were performed using a Transwell system (8-μm pore size; Corning Inc.). Briefly, 24 and 48 hours after siRNA transfection of Grb2 and control siRNA, cells were harvested and quantified. Next, 3 × 10^5^ cells were seeded onto the apical side of a Transwell chamber pre-coated with Matrigel (six-well insert) in serum-deprived culture media supplemented with 10% FBS was added to the basal compartment of the chamber to serve as a chemoattractant. The cells were allowed to migrate from top chamber to bottom chamber overnight for 24 hours and then fixed. The cells that remained on the apical side of the chamber were gently scraped off with cotton swabs. The invading cells were then quantified.

### Tube formation assay

RF-24 endothelial cells were plated on six-well plates at a density of 100,000 cells per well and allowed to attach overnight. The cells were transfected with siControl or siGrb2 at 24 hours. After 72 hours, cells were harvested and counted. A μ-plate for use in an angiogenesis assay was then coated with 10 μL of Matrigel, which was allowed to solidify at 37°C for 1 hour. Next, 20,000 cells per well were seeded on the Matrigel. The cells were incubated at 37°C for 6 hours. To assess endothelial cell tube formation, we counted and photographed complete tubes in randomly chosen fields at 40× magnification using an Olympus inverted microscope connected to a digital camera.

### *Ex-vivo* NMR metabolomics

Excised tumor tissue samples were flash frozen, weighed and crushed into fine powder in liquid nitrogen environment. These were immersed in 3 mL of methanol-to-water solution (2:1) and vortexed in presence of polymer beads. A rigorous process of mechanical homogenization was followed by centrifugation of the solution for ten minutes to separate the water-soluble metabolites from proteins and other cellular constituents [54]. Rotary evaporation method was used for the supernatant to remove the methanol. A lyophilizer was used to dry the sample out and collect the metabolites. The water soluble metabolites were finally dissolved in a solution of 600 μL of 2H_2_0, 36 μL of PO_4_ buffer, and 4 μL of 80 mM DSS (4,4-dimethyl-4-silapentane-1-sulfonic acid). Phosphate buffer was added to stabilize the pH variations, and DSS served as the reference standard to normalize the spectroscopic NMR signal of each metabolite [55].

NMR spectrum of each sample was obtained using a Bruker AVANCE III HD^®^ NMR scanner (Bruker Bio Spin Corporation, at room temperature. The operating frequency of the NMR spectrometer for proton resonance was 500 MHz and it was endowed with a triple resonance (^1^H, ^13^C, ^15^N) cryogenic temperature probe with a Z-axis shielded gradient. Water suppression sequence was employed using a pre-saturation technique of the RF pulse. Spectroscopic data were obtained with a 90° pulse width flowed, a scan delay t_rel_ of 6.0 s, and 1024 Hz spectral width. The time domain NMR signal was acquired using an exponential function. After the final spectrum was acquired, the phase correction was performed. Analysis of the metabolomics data was carried out using Chenomx NMR Suite 8.1 software (Chenomx Inc., Edmonton, Canada). Quantitative analysis of the metabolites was then performed using MestReNova software (Mestrelab Research, Spain) by integrating the resonance peak for each metabolite. Finally, the integral value was normalized by the value of the integral of the DSS resonance peak.

### Reverse phase protein array and pathway analysis

Reverse phase protein array (RPPA) assays were carried out by the MD Anderson Functional Proteomics RPPA Core. OVCAR8 cells were treated with siControl or siGrb2 for 72 hours. Cell lysates were then collected in RIPA buffer containing freshly added protease and phosphatase inhibitors. Protein concentrations were quantified using a BCA assay kit (Pierce Biotechnology), and 45 μg of protein from each group was used for RPPA analysis with a validated set of antibodies. To determine the biological function of Grb2, protein expression changes after siRNA transfection were used for pathway analysis with Ingenuity Pathway Analysis software and Netwalker pathway analysis software. The comparison analysis between siControl-treated cells and siGrb2-treated cells was carried out in R (version 3.5.1). Normalized data was at first log2 transformed (log2(x+1)). Differentially expressed proteins between the two groups were identified by a *p*-value, obtained from the moderated t-statistic from LIMMA package, of less than 0.05. To support visual data exploration, a heatmap for the most significant cases was generated using the heatmap.2 function from the gplots package.

### Statistical analysis

Student *t*-test (for comparison of two groups) and ANOVA (for comparison of all groups) were used to calculate *P* values for normally distributed data. Network and pathway analyses were performed using the NetWalker network analysis (version 1.0) and Ingenuity Pathway Analysis software programs. All statistical data were analyzed using the Prism software program (GraphPad Software). *P* values less than 0.05 according to two-tailed tests were considered significant. When multiple tests were performed, the BUM (beta uniform mixture) model [56] was used to fit *p*-values and estimate counts of significant features at different FDRs. All statistical tests were two-sided unless otherwise noted.

## SUPPLEMENTARY MATERIALS


