# Supplementary material for: Therapeutic efficacy of liposomal Grb2 antisense oligodeoxynucleotide (L-Grb2) in preclinical models of ovarian and uterine cancer
Source: Oncotarget. 2020 Jul 21;11(29):2819–33. doi: 10.18632/oncotarget.27667 (PMC7381098; doi:10.18632/oncotarget.27667)
Supplement: Supplementary file 1 [file oncotarget-11-2819-s001.pdf]

Therapeutic efficacy of liposomal Grb2 antisense oligodeoxynucleotide (L-Grb2) in preclinical models of ovarian and uterine cancer

SUPPLEMENTARY MATERIALS

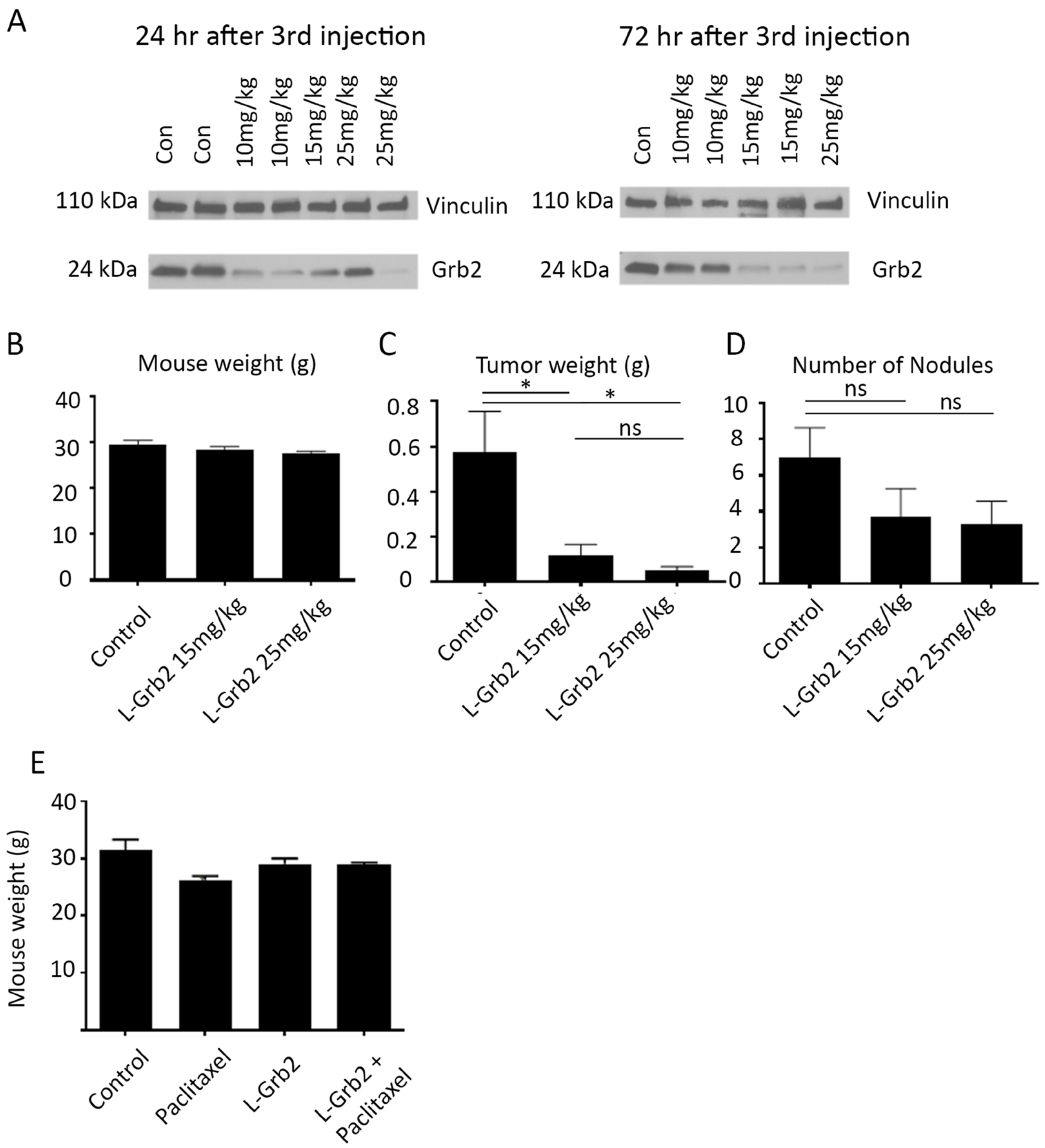

Supplementary Figure 1: Results of an L-Grb2 dose-defining experiment to evaluate ovarian tumor growth. (**A**) Western blot analysis of Grb2 protein expression in tumors obtained from mice inoculated with OVCAR5 cells 24 and 72 hours after the last intravenous injection of an empty DOPC liposome (control [Con]), 10 mg/kg L-Grb2, 15 mg/kg L-Grb2, or 25 mg/kg L-Grb2. (**B–D**) Mean body weights of (B), tumor weights in (C), and numbers of metastatic nodules in (D) mice intraperitoneally inoculated with OVCAR5 cells ($n = 10$ per group). Mice received treatment with L-Grb2 at 15 and 25 mg/kg twice weekly. (**E**) Mean body weights of mice inoculated with OVCAR5 cells that received control treatment, paclitaxel only (3 mg/kg) weekly, L-Grb2 only (15 mg/kg) twice weekly, or a combination of L-Grb2 and paclitaxel beginning 10 days after inoculation ($n = 9$ mice per group). Error bars, SEM. All statistical tests were two-sided. Asterisk indicates statistical significant of $^{*}p < 0.05$. NS indicates non-significant.

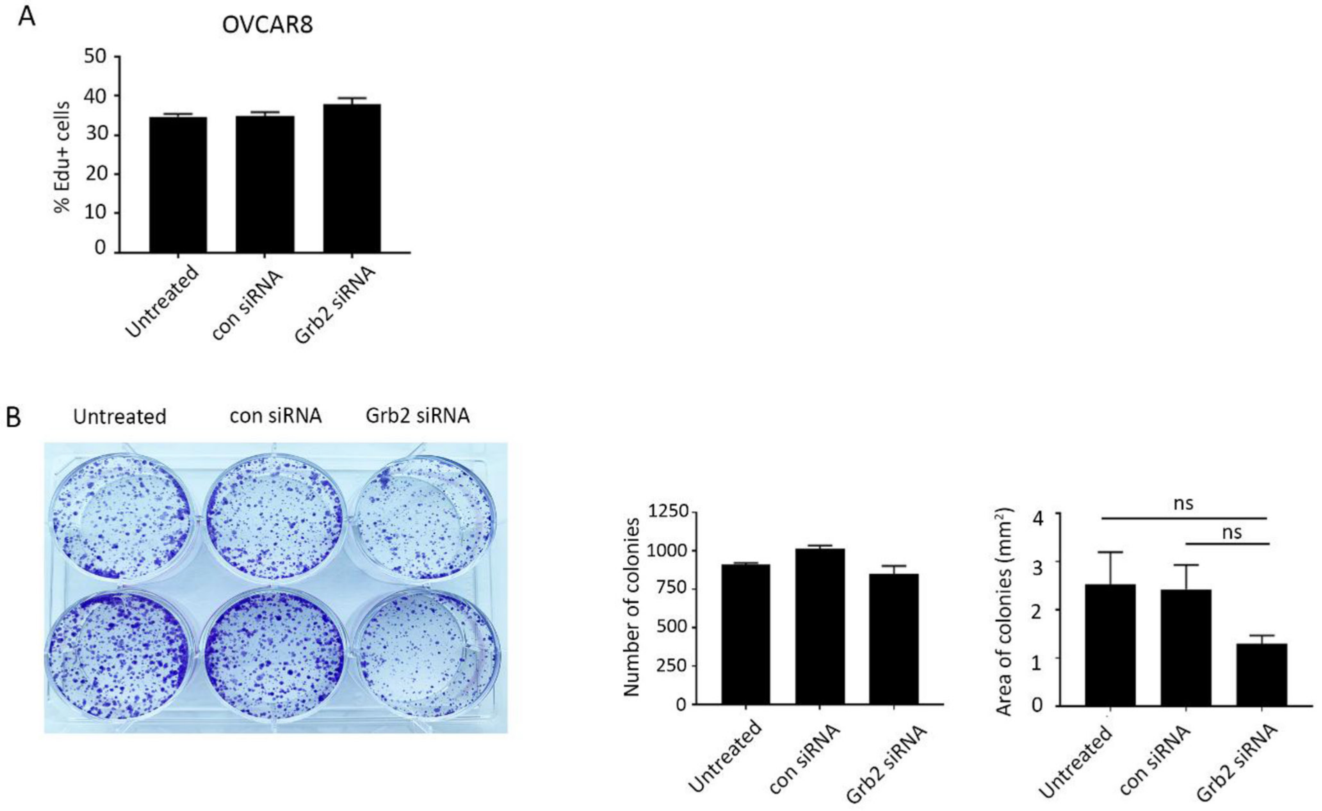

Supplementary Figure 2: Effect of Grb2 downregulation on ovarian cancer cell proliferation. (**A**) Results of an EdU incorporation assay performed to determine the number of proliferative untreated, siControl (con), and siGrb2 treated OVCAR8 cells (72 hours after treatment). (**B**) Results of a colony formation assay of the OVCAR5 cell line. After siRNA-based treatment, cell colonies were allowed to grow for 7–10 days before quantification. The adjoining graph shows the mean ± SEM total number of colonies and area of colonies in the three groups. NS indicates non-significant.

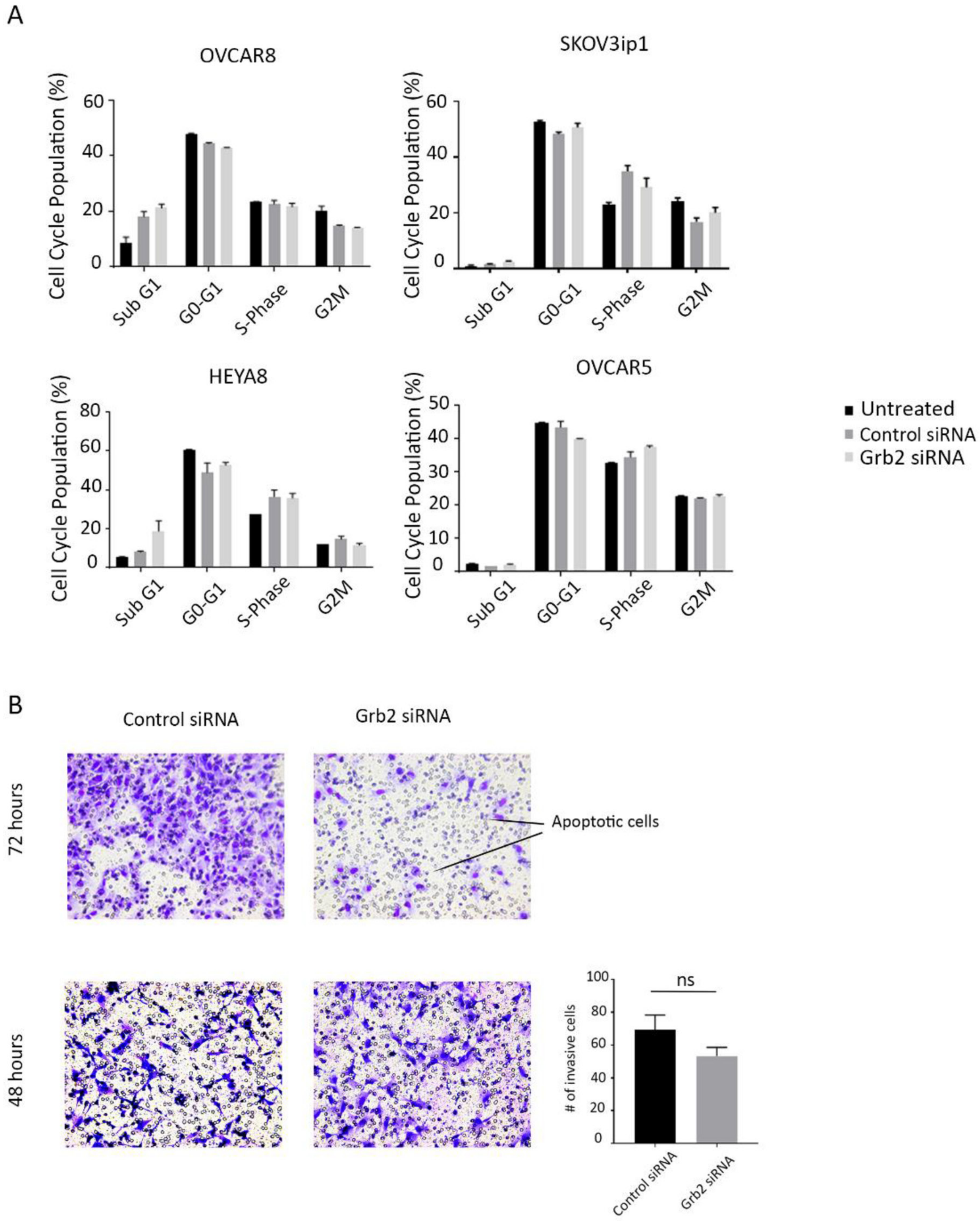

Supplementary Figure 3: Effect of Grb2 downregulation on cell-cycle progression and invasion in ovarian cancer cells. (**A**) Results of a cell-cycle assay performed using untreated, siControl-treated, and siGrb2-treated OVCAR8, SKOV3ip1, HEYA8, and OVCAR5 cells at 72 hours after transfection. (**B**) Results of a Matrigel invasion assay performed 48 and 72 hours after transfection of ovarian cancer cells with siControl or siGrb2. At 72 hours, cells were apoptotic, and quantification of invading cells was not performed. The adjoining graph shows the corresponding mean numbers of invasive cells 48 hours after transfection. Error bars, SEM. All statistical tests were two-sided. NS indicates non-significant.

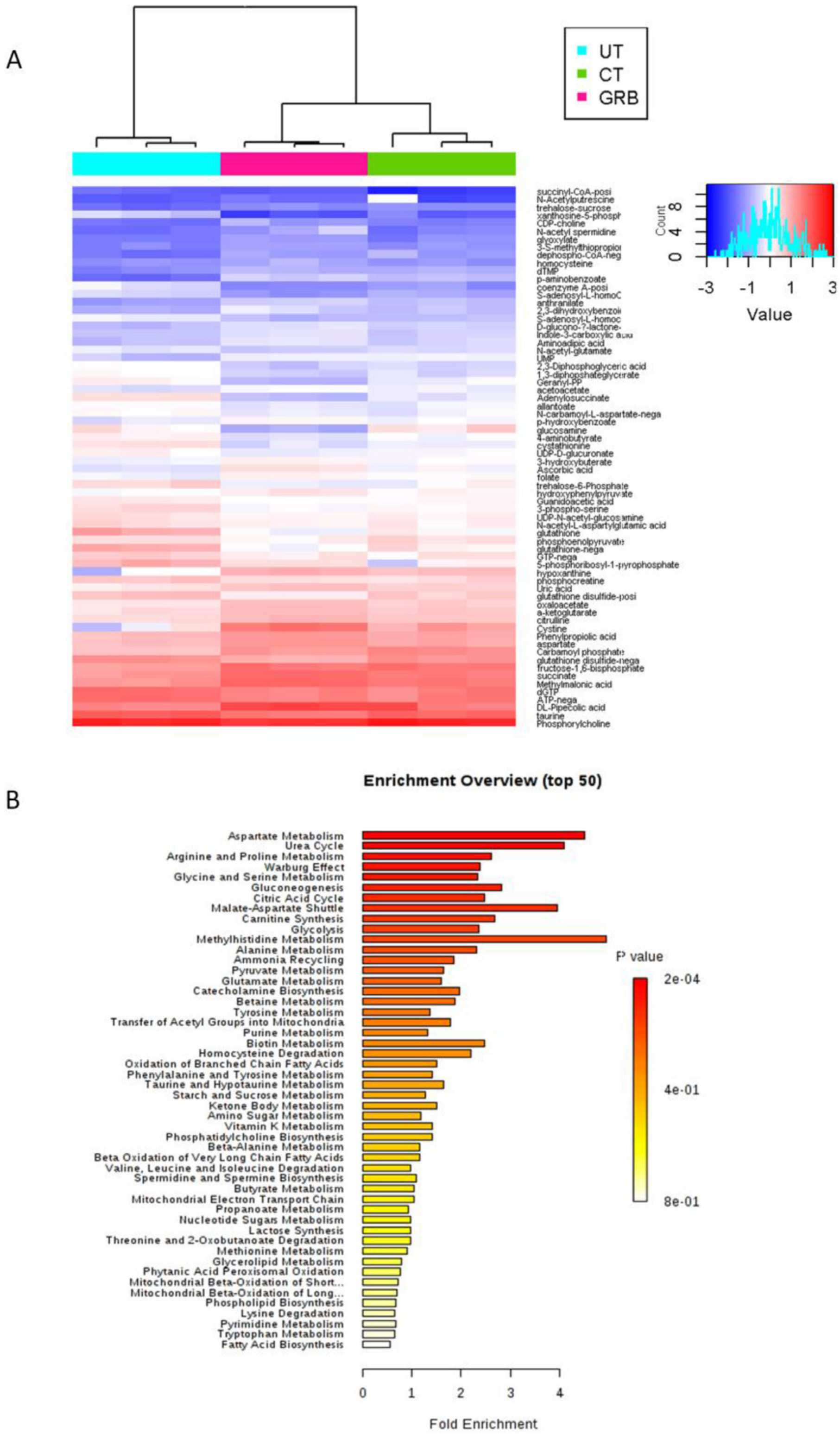

Supplementary Figure 4: Effect of Grb2 downregulation on metabolite levels in ovarian cancer cells. OVCAR8 ovarian cancer cells harvested after control or Grb2 siRNA transfection. Cells then underwent mass spectroscopy for quantification of metabolites. (**A**) Heatmap of 61 metabolites differentially expressed after Grb2 siRNA transfection. (**B**) Differential expression of pathways associated with metabolites in siGrb2-transfected but not in siControl-transfected ovarian cancer cells. Metabolite set enrichment analysis was performed using the MetaboAnalyst software program.

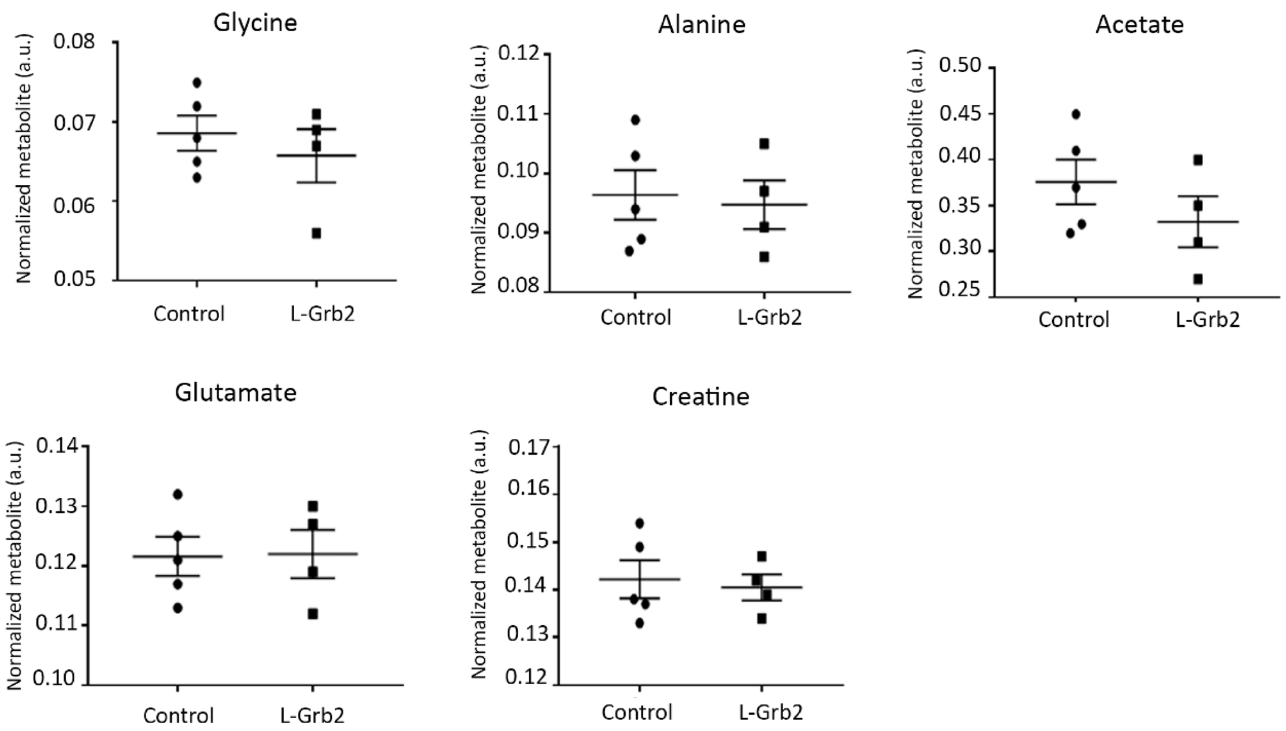

Supplementary Figure 5: Effect of Grb2 downregulation on mean metabolite levels in ovarian tumors. NMR spectroscopy was used to quantify metabolite levels in OVCAR5 tumors collected at the conclusion of an *ex vivo* therapeutic experiment from control mice and mice given L-Grb2–based monotherapy (n = 5). Error bars, SEM. All statistical tests were two-sided.

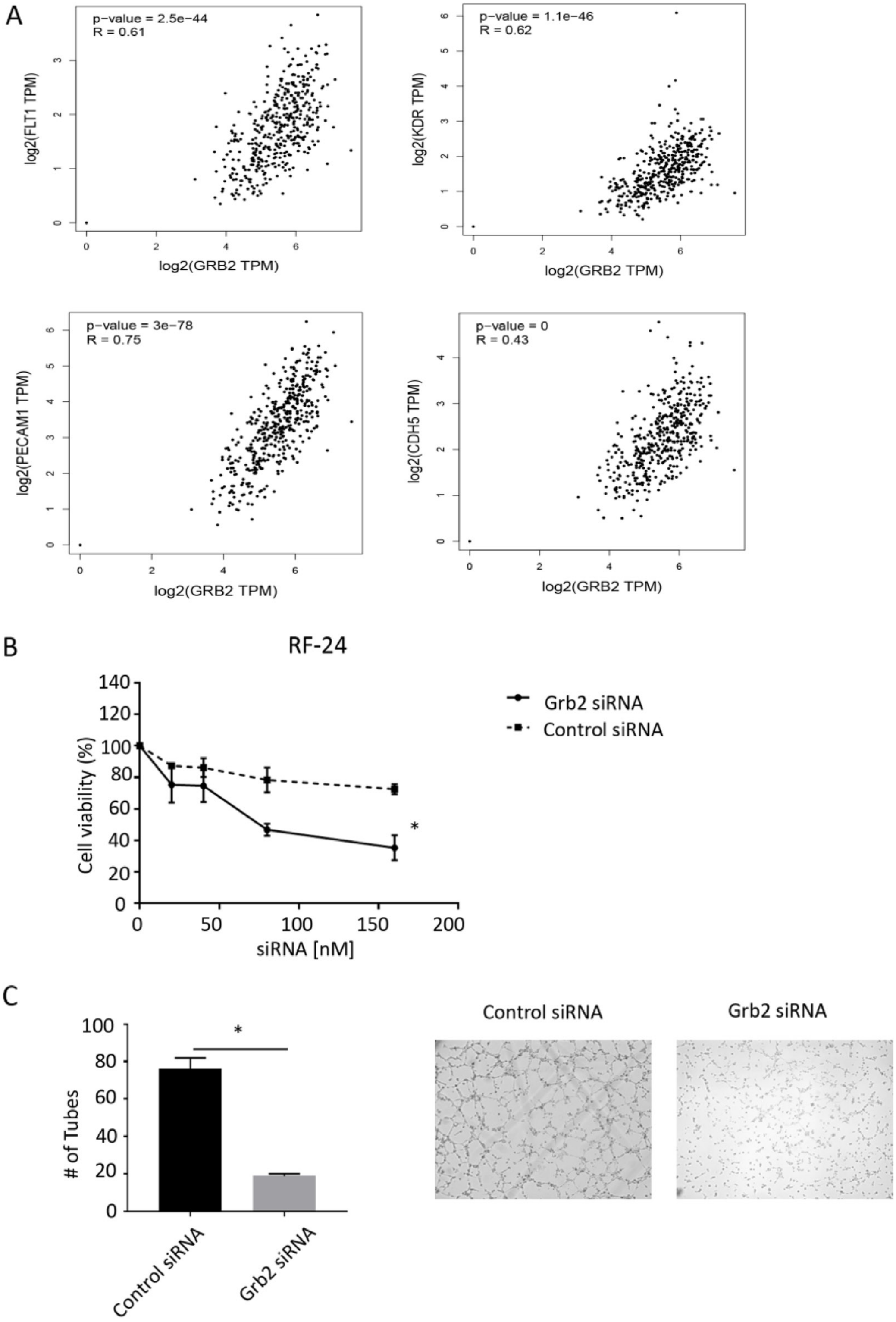

Supplementary Figure 6: (**A**) TCGA tumor mRNA seq expression data. Correlation between Grb2 expression and VEGFR1 (FLT1), VEGFR2 (KDR), PECAM1 and VE-Cadherin (CDH5). (**B**–**C**) *In vitro* effects of Grb2 downregulation on endothelial cells. (B) Results of an alamarBlue viability assay performed to determine the sensitivity of siGrb2-transfected RF-24 endothelial cells to Grb2 downregulation versus that of siControl-transfected RF-24 cells. The assay was performed 72 hours after transfection. The data represent the average values from triplicate measurements. (C) Left: the mean numbers of tubes formed by RF-24 cells on a gel matrix (n = 3 wells per group; mean number of tubes quantified using five pictures per well). Right: representative images of endothelial vessel formation of by RF-24 cells after transfection with siGrb2 or siControl. Images were taken at 40× magnification. Error bars, SEM. All statistical tests were two-sided. Asterisk indicates statistical significance of $^{*}p < 0.05$.

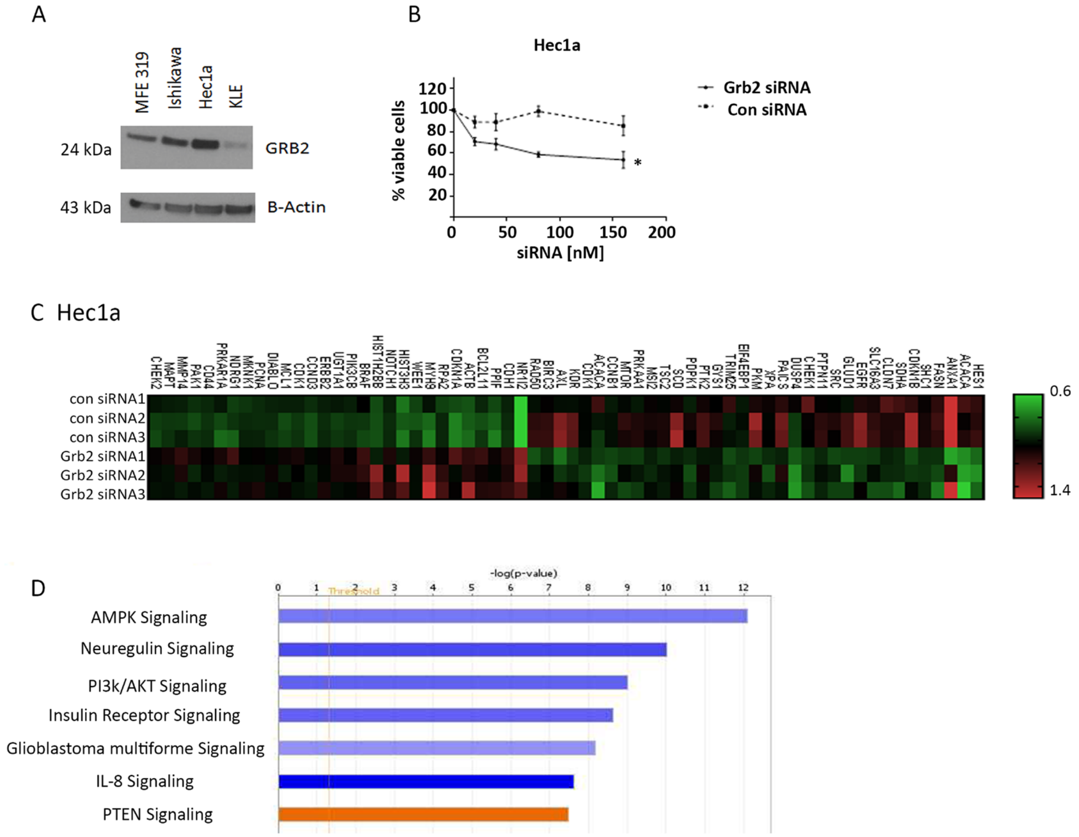

Supplementary Figure 7: Effect of Grb2 downregulation on uterine cancer cell lines. (**A**) Western blot analysis of Grb2 expression in a panel of uterine cancer cell lines. (**B**) An alamarBlue assay of Hec1a uterine cancer cells performed 72 hours after transfection to determine their percent viability. Data represent averages of triplicate measurements. Error bars, SEM. Asterisk indicates statistical significance of $^{*}p < 0.05$. (**C**) Differential expression of proteins in Hec1a cells after Grb2 downregulation as detected using an RPPA. Heat map of proteins whose expression differed significantly between siGrb2 and siControl transfected groups, $p < 0.05$. (**D**) Top canonical pathways altered after Grb2 downregulation using Ingenuity Pathway Analysis (IPA). All statistical tests were two-sided.

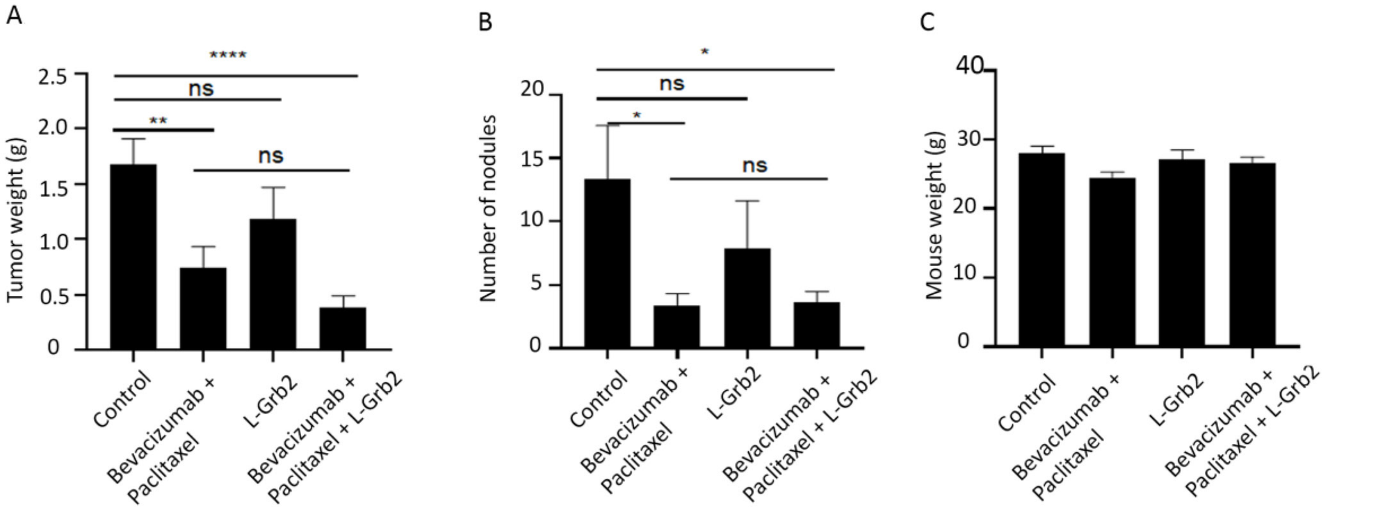

Supplementary Figure 8: Effects of L-Grb2 on uterine tumor growth. Mean tumor weights (**A**), number of metastatic nodules (**B**) and mouse weight (**C**) in mice inoculated with intrauterine injection of Hec1a cells that received an empty DOPC liposome (control), paclitaxel (3 mg/kg) weekly and bevacizumab (15 mg/kg) twice weekly, L-Grb2 (20 mg/kg) twice weekly or a combination of all three drugs beginning 10 days after inoculation ($n = 10$ mice per group). Error bars, SEM. All statistical tests were two-sided. NS indicates non-significant. $^{*}p < 0.05$, $^{**}p < 0.001$, $^{****}p < 0.0001$.

Supplementary Table 1: The networks most affected by Grb2 downregulation in ovarian cancer cells

Network	Effect	Number of occurrences*
Cellular response to hormone stimulus	Downregulated	10
Response to insulin stimulus	Downregulated	9
Negative regulation of apoptosis	Downregulated	8
Generation of precursor metabolites and energy	Downregulated	7
Regulation of leukocyte activation	Upregulated	10
Hematopoiesis	Upregulated	10
T-cell activation	Upregulated	7
Regulation of kinase activity	Upregulated	7

*The number of molecules significantly associated with the network.
